# Interrelation between Spectral Online Monitoring and Postoperative T1-Weighted MRI in Interstitial Photodynamic Therapy of Malignant Gliomas

**DOI:** 10.3390/cancers14010120

**Published:** 2021-12-27

**Authors:** Maximilian Aumiller, Christian Heckl, Stefanie Quach, Herbert Stepp, Birgit Ertl-Wagner, Ronald Sroka, Niklas Thon, Adrian Rühm

**Affiliations:** 1 Laser-Forschungslabor, LIFE Center, University Hospital, LMU Munich, 82152 Planegg, Germany; christianheckl1@web.de (C.H.); herbert.stepp@med.uni-muenchen.de (H.S.); ronald.sroka@med.uni-muenchen.de (R.S.); adrian.ruehm@med.uni-muenchen.de (A.R.); 2 Department of Urology, University Hospital, LMU Munich, 81377 Munich, Germany; 3 Department of Neurosurgery, University Hospital LMU Munich, 81377 Munich, Germany; Stefanie.Quach@med.uni-muenchen.de (S.Q.); Niklas.Thon@med.uni-muenchen.de (N.T.); 4 Department of Medical Imaging, The Hospital for Sick Children, University of Toronto, Toronto, ON M5G 1X8, Canada; birgitbetina.ertl-wagner@sickkids.ca

**Keywords:** malignant glioma, glioblastoma, photodynamic therapy, interstitial photodynamic therapy, 5-ALA, protoporphyrin IX, online monitoring, fluorescence, T1 hyperintensity, methemoglobin

## Abstract

**Simple Summary:**

Treatment monitoring is highly important for the delivery and control of brain tumor therapy. For interstitial photodynamic therapy (iPDT), an intraoperative spectral online monitoring (SOM) setup was established in former studies to monitor photosensitizer fluorescence and treatment light transmission during therapy. In this work, data from patients treated with iPDT as the initial treatment for newly diagnosed glioblastoma (*n* = 11) were retrospectively analyzed. Observed changes in treatment light transmission were assessed, and changes in optical tissue absorption were calculated out of these. In addition, magnetic resonance imaging (MRI) data were recorded within 48 h after therapy and showed intrinsic T1 hyperintensity in the treated area in non-contrast-enhanced T1-weighted sequences. A 3D co-registration of intrinsic T1 hyperintensity lesions and the light transmission zones between cylindrical diffuser fiber pairs showed that reduction in treatment light transmission corresponding to increased light absorption had a spatial correlation with post-therapeutic intrinsic T1 hyperintensity (*p* ≤ 0.003).

**Abstract:**

In a former study, interstitial photodynamic therapy (iPDT) was performed on patients suffering from newly diagnosed glioblastoma (*n* = 11; 8/3 male/female; median age: 68, range: 40–76). The procedure includes the application of 5-ALA to selectively metabolize protoporphyrin IX (PpIX) in tumor cells and illumination utilizing interstitially positioned optical cylindrical diffuser fibers (CDF) (2–10 CDFs, 2–3 cm diffusor length, 200 mW/cm, 635 nm, 60 min irradiation). Intraoperative spectral online monitoring (SOM) was employed to monitor treatment light transmission and PpIX fluorescence during iPDT. MRI was used for treatment planning and outcome assessment. Case-dependent observations included intraoperative reduction of treatment light transmission and local intrinsic T1 hyperintensity in non-contrast-enhanced T1-weighted MRI acquired within one day after iPDT. Intrinsic T1 hyperintensity was observed and found to be associated with the treatment volume, which indicates the presence of methemoglobin, possibly induced by iPDT. Based on SOM data, the optical absorption coefficient and its change during iPDT were estimated for the target tissue volumes interjacent between evaluable CDF-pairs at the treatment wavelength of 635 nm. By spatial comparison and statistical analysis, it was found that observed increases of the absorption coefficient during iPDT were larger in or near regions of intrinsic T1 hyperintensity (*p* = 0.003). In cases where PpIX-fluorescence was undetectable before iPDT, the increase in optical absorption and intrinsic T1 hyperintensity tended to be less. The observations are consistent with in vitro experiments and indicate PDT-induced deoxygenation of hemoglobin and methemoglobin formation. Further investigations are needed to provide more data on the time course of the observed changes, thus paving the way for optimized iPDT irradiation protocols.

## 1. Introduction

The standard of care for treating newly diagnosed malignant gliomas comprises surgical resection, when feasible, followed by radiochemotherapy. Unfortunately, the median survival for patients suffering from high grade gliomas continues to be low [1,2]. In an attempt to improve this dismal prognosis, the feasibility of photodynamic therapy (PDT) has been investigated since the 1980s [3,4]. So far, several clinical trials using different photosensitizers have been published (for an overview, see [5]). Several clinical studies have explored the use of 5-aminolevulinic acid (5-ALA) as a photosensitizer precursor [6,7,8,9,10], which has some intriguing advantages over other photosensitizers for treating high grade gliomas:

- There is much less concern about unspecific photosensitization during circulation and tissue distribution, as 5-ALA itself is not photoactive [11].
- Clinically approved, 5-ALA is also widely used for protoporphyrin IX (PpIX)-based fluorescence-guided resection of malignant gliomas [11,12,13].
- The photosensitizer PpIX is produced in the mitochondria of malignant glioma cells with a high selective accumulation compared to adjacent tissue [11].
- Due to the relatively fast photobleaching of PpIX, any low PpIX concentration metabolized in adjacent tissue will be used up before cell death is induced [14,15,16].

There are two different approaches to deliver 5-ALA-based PDT for malignant gliomas: as an adjuvant treatment after surgical resection in order to destroy residual PpIX-containing diffusely infiltrating glioma cells [17,18] or as a primary treatment in a stereotactic interstitial approach by inserting cylindrical diffuser fibers (CDF) into the glioma tissue, named interstitial PDT (iPDT) [6,10,14].

In any approach, sufficient light needs to be delivered throughout the relevant tissue volumes. Light penetration depends on the wavelength-dependent optical tissue parameters governing light absorption and scattering. Utilizing Monte Carlo simulations, it was shown that inside the tissue the light fluence rate around a CDF used during iPDT typically decays exponentially to 1/e (37%) within less than 3 mm for red light [6,19]. At 635 nm, the iPDT treatment wavelength used for excitation of the 5-ALA-induced PpIX, blood is still the dominant absorber. Therefore, the absorption depends, critically, also on the blood oxygenation level, as the absorption coefficient of deoxygenated blood is about 8.5 times higher than that of oxygenated blood at this specific wavelength [20]. As PDT consumes tissue oxygen, the oxygenation status of hemoglobin—and thus its absorption coefficient—may dynamically vary during treatment as reported [21,22]. It can be hypothesized that intracerebral hemorrhages induced by the implantation procedure may, therefore, have a rather complex impact on treatment success. To elucidate the involved processes, the effects of 5-ALA-PDT on hemoglobin absorption were experimentally assessed in an artificial liquid tissue phantom model in a prior study [23]. In artificial samples containing intact erythrocytes, almost no change in optical parameters was observed when the illumination started and PpIX photobleaching occurred continuously. If lysed erythrocytes were used in the liquid phantom, however, a change in treatment light transmission was observed immediately after initiating treatment. Comparing these two experiments shows that some time is needed for the erythrocyte cell membrane to rupture under laser irradiation, plus further time for the hemoglobin to be deoxygenated. Once the erythrocyte cell membrane ruptured, rapid deoxygenation of the hemoglobin occurred, and, in addition, met-hemoglobin (MetHb) was formed. As the absorption of deoxy-hemoglobin (Hb) and, even more so, of MetHb at 635 nm is much stronger than the absorption of oxy-hemoglobin (HbO_2_) (about 8.5 and 33 times higher, respectively), this has an immediate effect on the light distribution [23].

Clinically, intercranial hemorrhages are visualized and controlled using CT and MRI. In case of an intracerebral hemorrhage, the MRI signal characteristics depend on the cellular location and the different types of hemoglobin produced during the breakdown and removal of blood [24]. Typically, in the acute setting after intracerebral hemorrhage, intracellular HbO_2_ or, later, Hb appears largely isointense to the surrounding brain parenchyma in non-enhanced T1-weighted MR imaging. Two to three days after PDT illumination, the formation of intracellular MetHb is usually seen as intrinsic signal hyperintensity on T1-weighted images [24,25,26,27,28]. Whether and how these changes are seen after PDT treatment, and whether and how they may be related to the PDT procedure, has not been systematically investigated so far.

Therefore, in this investigation, intraoperative changes in treatment light transmission and PpIX fluorescence are assessed, based on measurements before and after iPDT according to the spectral online monitoring (SOM) procedure described in [7,10,29], retrospectively analyzed for a number of patients with high grade glioma undergoing iPDT [8]. A further aim was the investigation of potential relations between changes in light absorption within the tissue and intrinsic T1 hyperintensity visible on early postoperative MRI within 48 h after treatment. To this end, the appearance, strength, as well as 3D positional relations of increased light absorption within the tissue and intrinsic T1 hyperintensity in early postoperative MRI data were analyzed.

## 2. Materials and Methods

### 2.1. Data Acquisition

**Figure 1:**
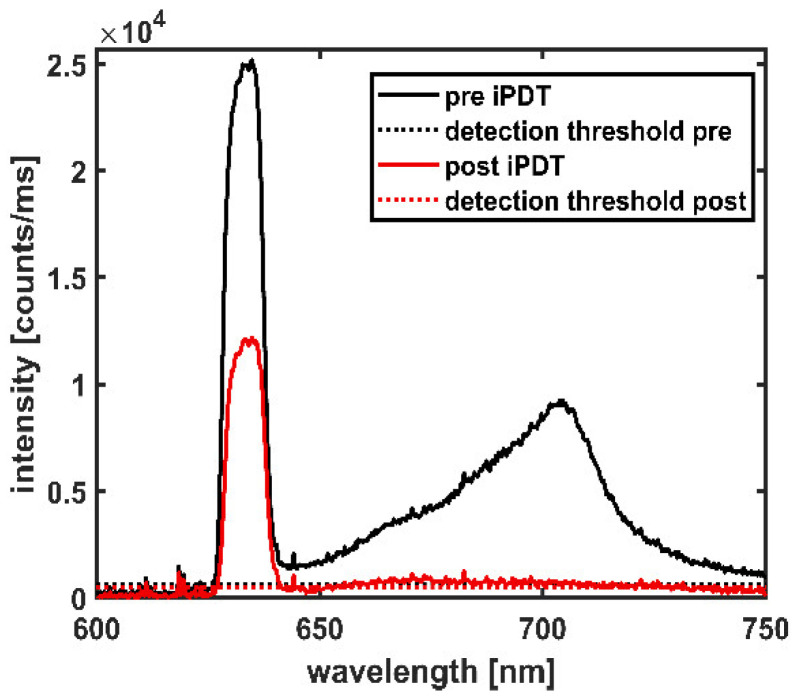
Spectral online monitoring data of a CDF-pair shown at iPDT start (pre iPDT, solid black line) and iPDT end (post iPDT, solid red line). Dotted lines indicate the detection thresholds.

Ethical approval for the retrospective analysis of the collected data was obtained from the institutional review board (University Hospital LMU Munich ethics protocol: UE no. 335–16). SOM and MRI data were obtained from patients suffering from de novo GBM treated with salvage iPDT [8] and were analyzed retrospectively (*n* = 11; 8/3 male/female; median age: 68, range: 40–76). Relevant irradiation and spectral online monitoring characteristics are listed in Table 1. In one case, the entire tumor could not be treated in one single treatment session due to its localization. Therefore, iPDT was applied in two successive treatment sessions: iPDT cases #4a and #4b. The iPDT treatment was performed as earlier described [6,8]. The number of CDFs and the insertion coordinates for stereotactic placement of the fibers were determined by careful 3D treatment planning (target 1.19 software, Brain LAB AG, Munich, Germany), with the number of CDFs employed per iPDT ranging from 2 to 10. Parallel placement of the CDFs is favored but not always possible due to the shape of the target volume or other restrictions to be considered during the treatment planning. Treatment light with a wavelength of 635 nm was delivered by a 4-port diode laser system (Ceralas PDT Diode Laser, biolitec AG, Jena, Germany). The CDFs (outer diameter 1.56 mm; Light Guide Optics, Rheinbach, Germany) with a 20 or 30 mm diffuser length were connected to the laser ports via short connecting fibers. To perform SOM measurements, all treatment fibers, except the one for illumination and excitation for the desired measurement, were temporarily disconnected from the short connecting fibers and instead connected to the inputs of a fiber switch (MPM-2000, Mikropack, Ostfildern, Germany) [10,29]. The output of the fiber switch was guided to a spectrometer (USB2000+, Ocean Insight, former Ocean Optics, Ostfildern, Germany) with a long pass inline filter (RG645, Schott Glas, Mainz, Germany) at the spectrometer entrance. PDT illumination was conducted with a 200 mW/cm diffuser length for 1 h as standard-setting [30], if not indicated otherwise in Table 1. It was shown that, for comparable scenarios, this power setting limits the tissue heating to below 42 °C [6]. A minimum target light dose of 18.7 J/mm^2^ was used for treatment planning, as previously calculated based on the concept of “complete” photobleaching [31]. In cases where interfiber distances below 9 mm had to be chosen due to restrictions in the treatment planning (e.g., blood vessels near the trajectories), the power was reduced and the irradiation time prolonged to prevent thermal damage. SOM was performed twice, immediately before (pre) and after (post) iPDT illumination, thus receiving the pre and post iPDT spectra, exemplarily illustrated in Figure 1. The transmitted treatment light is visible in the wavelength range of 626–642 nm. The PpIX fluorescence signal is in the range of 650–750 nm. In total, 320 spectra (pre plus post iPDT) were recorded and analyzed (range: 2–36 per iPDT case, median: 24), representing about 41% of all possibilities of measurable spectra (784 for 392 CDF-pair combinations).

### 2.2. Spectral Data Assessment

The spectra measured during the SOM procedure were assessed regarding treatment light transmission around 635 nm and PpIX fluorescence around 705 nm (see Figure 1). The transmitted treatment light was analyzed concerning its integral signal intensity *I*_t_ (in counts/ms), recorded within the wavelength interval of 626–642 nm according to Equation (1).

(1) $$I_{t}=\overset{642\;nm}{\underset{626\;nm}{\int }}I\left(\lambda \right)\;d\lambda \;$$

where *I*(*λ*)d*λ* is the intensity measured within a wavelength interval d*λ*. The PpIX fluorescence was assessed by fitting a normalized pure PpIX fluorescence spectrum *I*_PpIX_(*λ*) and a normalized auto-fluorescence spectrum *I*_auto_(*λ*) to the recorded data *I*_rec_(*λ*) in the wavelength range of 650–750 nm:

(2) $$I_{\mathrm{rec}}\left(\lambda \right)=a\;I_{\mathrm{PpIX}}\left(\lambda \right)+b\;I_{\mathrm{auto}}\left(\lambda \right)\;$$

with adjustable weighting factors *a* and *b*. The first addend in Equation (2) represents the fitted PpIX spectrum contained in the recorded spectra. This fluorescence was analyzed based on its maximum signal intensity value *I*_f_ (in counts/ms) recorded within the wavelength range 702–708 nm:

(3) $$I_{f}=\max\left\{a\;I_{\mathrm{PpIX}}\left(\lambda \right)\;|\;702\;\mathrm{nm}\leq \lambda \leq 708\;\mathrm{nm}\right\}\;$$

The signal intensities *I*_t_ and *I*_f_ given by Equations (1) and (3) were considered detectable (above detection threshold) if the maximum signal intensities *I*(*λ*) within the specified wavelength intervals were higher than three times the noise level, which was determined individually for each spectrum from the spectral range of 500–550 nm, outside the ranges of transmitted treatment light and fluorescence. To obtain a measure for the change of treatment light transmission during iPDT, the intra-operative pre versus post iPDT treatment light transmission ratio *R*_pre/post_ is calculated using Equation (4):

(4) $$R_{\mathrm{pre}/\mathrm{post}}=\frac{I_{t,\mathrm{pre}}}{I_{t,\mathrm{post}}}$$

If *I*_t,pre_ was below the detection threshold, *R*_pre/post_ was defined as 0. Multiple measurements on tissue phantoms showed that the statistical uncertainty of the signal intensities *I*_t_ and *I*_f_ given by Equations (1) and (3) was not more than 8%. Propagation of uncertainty with the variance formula yields an uncertainty <20% for *R*_pre/post_.

### 2.3. Calculation of Intensities and Optical Tissue Properties

Diffusion approximation was used for all calculations to derive the absorption coefficient µ_a_ of the tissue at the treatment wavelength of 635 nm using the measured treatment light transmission signal intensities *I*_t_ between the CDF-pairs. It was assumed that the CDFs emit and detect light homogeneously. The active CDF-sections were approximated as linear arrays of isotropically emitting point sources or isotropically collecting point detectors (five sources or detectors per mm diffuser length, each contributing with the same weight). The photon fluence rate *Φ_ij_* generated for each photon emitted from one point source *i* on the emitting CDF at the location of one point detector *j* on the detecting CDF is given by [32,33]:

(5) $$\Phi_{ij}\left(r_{ij}{,\;\mu }_{a}{,\;\mu }_{s}^{\prime }\right)=\frac{1}{4\;{\pi \;r}_{ij}}\;3\left(\mu_{a}+\mu_{s}^{\prime }\right)\;\exp\left(-r_{ij}\;\sqrt{3\mu_{a}\left(\mu_{a}+\mu_{s}^{\prime }\right)}\right)$$

where *r_ij_* is the distance between an emitting and a detector point and μ_s_’ the reduced scattering coefficient of the tissue. The double sum over Equation (5) for all emitting points *i* and detector points *j* includes all point-to-point combinations between the two CDFs and is proportional to the theoretically expected value of the transmitted treatment light intensity *I*_t,theo_ for the CDF-pair [34]. As for constant emission power per active CDF length, the total power emitted by a CDF scales with the length of its light-emitting section; *L*_E_, *I*_t,theo_ is described by Equation (6):

(6) $$I_{t,\mathrm{theo}}=\frac{P_{0}\cdot L_{E}}{N}\underset{i}{\sum }\underset{j}{\sum }\Phi_{ij}\left(r_{ij},\mu_{a}{,\mu }_{s}^{\prime }\right)\;$$

where *N* is the number of point combinations. The constant calibration factor *P*_0_ (in cm∙counts/ms) includes the emitted photon rate per cm diffuser length and the conversion factor from photon fluence rate at the detecting CDF-section to measured intensity. To note, Equation (6) is independent of the length of the active CDF section of the detector, which relies on the assumptions that the detection efficiency is homogeneous and the total detection efficiency is independent of the diffuser length. *P*_0_ was determined experimentally by fitting Equation (6) to transmitted laser intensities acquired from measurements with the iPDT SOM setup in liquid artificial tissue phantoms with pre-characterized optical properties made from ink (brilliant black, Pelikan 4011, Pelikan Group GmbH, Berlin, Germany), Lipovenös^®^ (Lipovenös MCT 20%, Fresenius, Bad Homburg, Germany), and distilled water. The measurements were performed between CDF-pairs with various active diffuser lengths in a distance-dependent manner.

To determine an average absorption coefficient μ_a_ at 635 nm for the tissue volume surrounding the CDF-pair in the clinical case, the integral transmitted treatment light intensity *I*_t_ obtained from the spectral measurements is compared to *I*_t,theo_(μ_a_,μ_s_’) (see Equation (6)). During this comparison, the absorption coefficient μ_a_ in Equation (6) is adjusted, and the reduced scattering coefficient μ_s_’ is kept constant at $2{\;\mathrm{mm}}^{-1}$ [6]. The distance values *r*_ij_ inserted in Equations (5) and (6) were calculated using the coordinates of the active CDF-sections defined in the treatment plan. These coordinates further define the coordinates of the emitter and detector points of each CDF-pair. From these calculations, the minimum distance was derived as a distance classifier for each CDF-pair, as the CDFs often cannot be placed perfectly in parallel to each other. For each CDF-pair, a lookup table of intensity values *I*_t,theo_(μ_a_) according to Equation. (6) was calculated for a set of μ_a_ values from 0.0001 to 0.75 mm^−1^ with a resolution of 0.0001 mm^−1^. The μ_a_ value leading to the best match between the calculated intensity *I*_t,theo_(μ_a_) and the intraoperatively measured intensity *I*_t_ was selected and defined as the representative absorption coefficient for the tissue volume surrounding the specified CDF-pair.

For CDF-pairs with no transmission (transmitted treatment light intensity below detection threshold) pre or post iPDT, the minimally needed μ_a_ to obtain a signal intensity equal to the detection threshold was determined, using the same procedure. From the μ_a_ values obtained from the pre and post iPDT spectra, the difference Δμ_a_ induced during the iPDT illumination was calculated. For iPDT case #1, Δμ_a_ was calculated based on *R*_pre/post_, but not the two μ_a_ values themselves, because an individual optical filter was used, which was different from the filter used for the SOM procedure in all other iPDT cases, so the predetermined *P*_0_ value was not suitable.

In addition, forward calculations were performed with Equation (6) to examine the influence of different blood volume fractions (bvf) and hemoglobin species in tumor tissue on *I*_t_. As blood is the main absorber in tissue at 635 nm [35], the μ_a_ values inserted into Equation (6) were set to those of the respective hemoglobin species multiplied with the assumed bvf, while the absorption of all other tissue constituents was assumed to be neglectable. The following absorption coefficients at 635 nm were used for the different pure hemoglobin species: HbO_2_: μ_a_ = 0.2477 mm^−1^; Hb: μ_a_ = 2.1149 mm^−1^ and MetHb: μ_a_ = 8.1073 mm^−1^ [20,36]. The average oxygen saturation of normal capillary blood is assumed to be 85% [37]. Therefore, a mixture of 85% HbO_2_ and 15% Hb is used in the calculations for this case. To sum up, this resulted in the following absorption coefficients of the tissue per % bvf: 0.0053 mm^−1^/% for capillary blood, 0.0211 mm^−1^/% bvf for Hb, and 0.0811 mm^−1^/% bvf for MetHb.

### 2.4. CT and MRI-Protocol

A contrast-enhanced CT was acquired for treatment planning after the induction of anesthesia and assembly of the stereotactic frame. MR images were acquired within 14 days before iPDT for treatment planning and on the day after iPDT for treatment assessment (median: 22 h after the end of therapy; range: 15–29 h). Pre- and postoperative MRI was performed with a GE SIGNA HDxt 3T scanner (GE Healthcare, General Electric, Chicago, IL, USA). T1-weighted sequences (Repetition time TR = 6.5 ms; Echo time TE = 3.15 ms) were recorded before (3D; resolution 0.4297 × 0.4297 × 3.5 mm^3^) and after (3D; resolution 0.4297 × 0.4297 × 0.7 mm^3^) administration of a Gadolinium-based contrast agent (GBCA).

### 2.5. MRI Analysis

By affine transformation using mutual information, MR images were co-registered with the CT scan in the stereotactic frame [38]. For this purpose, all images were resampled to the lowest image resolution (0.4297 × 0.4297 × 0.5 mm^3^) via 3D extrapolation. The resampling and automatic co-registration of the MR and CT images were performed using Advanced Normalization Tools (ANTs; version 2.3.2, University of Pennsylvania, Philadelphia, PA, USA, University of Virginia, Charlottesville, VA, USA and University of Iowa, Iowa City, IA, USA) [39]. The co-registration was visually verified and, if necessary, adjusted manually using ITK-Snap (version 3.8, University of Pennsylvania, Philadelphia, USA) [40]. The tumor volume was defined in the pre-therapeutic contrast-enhanced T1-weighted MR images. The T1 hyperintensity structure was segmented in the post-therapeutic non–contrast enhanced T1-weighted MRI. T1 hyperintensity structures were defined as regions where the intensity was higher than in the directly surrounding tissue in the post-therapeutic dataset but not in the pre-therapeutic dataset. The segmentation of all structures was performed manually and was independently reviewed by two medical doctors (S.Q., neurosurgeon with more than 5 years experience; and B.E-W., neuroradiologist with more than 15 years of post-fellowship experience in interpreting MRI). To characterize the spatial relation of the tumor volume (V_T_) and the intrinsic T1 hyperintensity (V_hyp_) structures, the Dice similarity coefficient (DSC, Equation (7)), and the Jaccard similarity coefficient (JSC, Equation (8)), as well as the overlap volume (OV, Equation (9)), were calculated [41,42,43,44]).

(7) $$\mathrm{DSC}=\frac{2\;\left|V_{T}\cap V_{\mathrm{hyp}}\right|}{\left|V_{T}\right|+\left|V_{\mathrm{hyp}}\right|}$$

(8) $$\mathrm{JSC}=\frac{\left|V_{T}\cap V_{\mathrm{hyp}}\right|}{\left|V_{T}\cup V_{\mathrm{hyp}}\right|}$$

(9) $$\mathrm{OV}=\frac{\left|V_{T}\cap V_{\mathrm{hyp}}\right|}{\min\left(\left|V_{T}\right|,\left|V_{\mathrm{hyp}}\right|\right)}$$

A DSC or JSC value of 1 indicates identical volumes in shape, localization, and size. An OV of 1 indicates that the smaller volume is fully included in the larger volume. DSC, JSC, and OV values of 0 all indicate disjunct volumes without any overlap. In addition to the overlap parameters, the volume ratios V_hyp_/V_T_ and (V_T_ ∩ V_hyp_)/V_T_ were calculated.

For the intrinsic T1 hyperintensity analysis, a normalized value of the MRI signal intensity within the intrinsic T1 hyperintensity region (I_T1_) is calculated. I_T1_ provides at least a rough estimate of the degree of T1 hyperintensity. It further considers different gain settings of the images, allowing comparability between the different iPDT cases.

(10) $$I_{T1}=\frac{{Ī}_{T1}}{{Ī}_{WM}}$$

According to Equation (10), I_T1_ is defined as the ratio of the mean image intensity within the intrinsic T1 hyperintensity ( Ī_T1_) and the mean image intensity of white matter regions (Ī_WM_). For this purpose, seven isotropic spheres (diameter 8 mm) containing only white matter were drawn in the brain hemisphere unaffected by the tumor, and their overall mean image intensity was calculated. In this calculation, background intensity was neglected, as it was less than 1% of the image intensity.

### 2.6. Overlap Calculation between T1 Hyperintensity and CDF-Pairs

**Figure 2:**
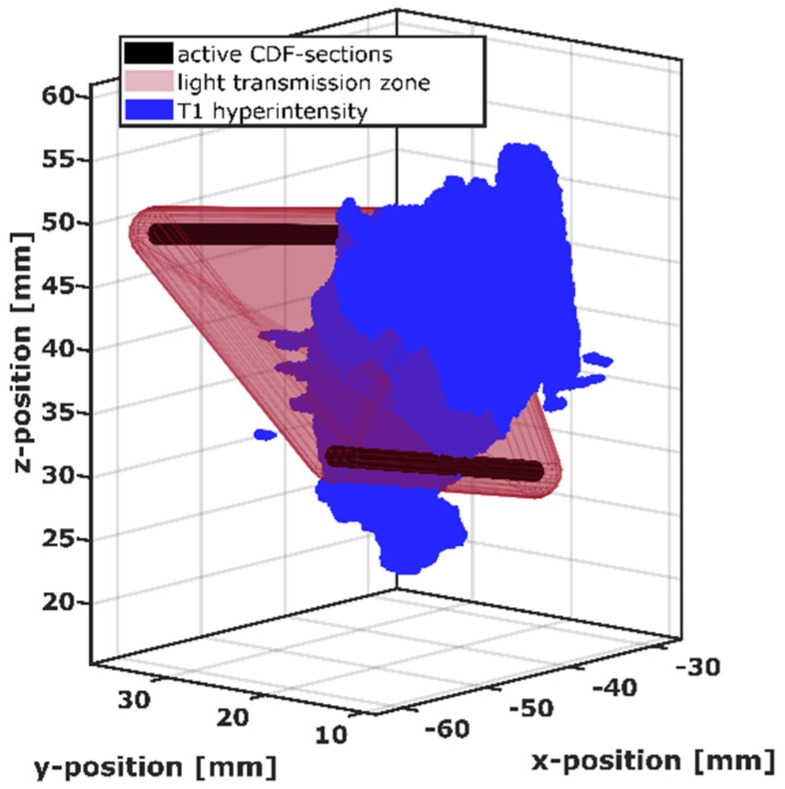
Visualization of the intersection between the light transmission zone (red) of the active cylindrical diffuser fiber (CDF) sections of one CDF-pair (black) and the intrinsic T1 hyperintensity volume (blue).

For further assessment of the obtained MRI and SOM results, the spatial relation between intrinsic T1 hyperintensity and the positions of the CDFs placed in the tumor was examined. To determine whether the T1 hyperintensity is located between a CDF-pair and how much it may have affected the light transmission, a 3D volume of simple geometrical shape (see Figure 2) was defined around the active sections of each CDF-pair. That volume roughly represents the region in which most light propagation occurs from one to the other CDF. This “light transmission zone” is a volume with standardized shape which was intersected with the intrinsic T1 hyperintensity, as shown in Figure 2. For the positional analysis, the coordinates of the stereotactic CDF trajectory were transformed to CT image coordinates, using a linear coordinate transformation, and the image coordinates of the active CDF-section along the trajectory were determined. Based on these coordinates, the predominant light transmission zone surrounding a CDF-pair was defined by fluence calculations between two parallel line sources placed at a 10 mm distance. Assuming tumor-like tissue optical properties (μ_a_ = 0.02 mm^−1^, μ_s_’ = 2 mm^−1^ [6]), Equations (5) and (6) were used to determine the two light fluence rates that would be generated in every voxel (size 0.1 × 0.1 × 0.1 mm^3^) of a large discretized volume surrounding the CDF-pair if one or the other active CDF-section would emit light, respectively. By multiplication of these fluence rates (Equation (11)), an estimate measure for the contribution of a voxel with coordinates (x,y,z) to the treatment light transmission signal expected for this CDF-pair is obtained.

(11) $$I_{\mathrm{voxel}}\left(x,y,z\right)\;\sim \;\Phi_{1}\left(x,y,z\right)\;\cdot \;\Phi_{2}\left(x,y,z\right)$$

Using this estimation, the light transmission zone was defined by combining the voxels with the strongest contributions, up to a total contribution of 2/3 (67%) compared to the sum over all voxels. The size of this combined volume corresponds to a convex hull surrounding two capsules of radius 2.1 mm constructed around the two active sections of the CDF-pair. The definition of the light transmission zone was standardized in this way for all CDF-pairs, i.e., by the construction of capsule-shaped volumes with radius 2.1 mm around the active CDF-sections and the subsequent construction of the convex hull around these two capsule-shaped volumes. For the 3D analysis, the capsule-shaped volumes are built up employing a computer aided design program (FreeCAD version 0.18.16117 © Juergen Riegel, Werner Mayer, Yorik van Havre 2001–2019), and the convex hull around them is computed automatically with Matlab R2018b (MathWorks Inc., Natick, MA, USA) during the intersection process. For the positional analysis of the volumes illustrated in Figure 2, the T1 hyperintensity volume was transformed into a tetrahedral mesh with an average tetrahedron size of 0.02 mm³ (min: 0.0004 mm³, max: 0.06 mm³) using a mesh tool based on the computational geometry algorithms library (CGAL) [45]. For each tetrahedron, it was checked whether it was within the light transmission zone of a CDF-pair, and the overlap volume was determined by summing those tetrahedron volumes. In case of a non-zero overlap volume, the CDF-pair was regarded as affected by T1 hyperintensity.

### 2.7. Data Evaluation and Statistics

Descriptive p-values were calculated using the Mann–Whitney U test and the two-sided Spearman test (alpha = 0.05; power = 0.8). Statistical comparisons were performed using IBM SPSS Statistics for Windows, version 25 (IBM Corp., Armonk, NY, USA). Mathematical calculations and data fitting procedures were implemented in MATLAB R2018b (MathWorks Inc., Natick, MA, USA). The 3D visualizations and further 3D analysis were completed using Paraview version 5.6.0 (Kitware, Inc., Clifton Park, NY, USA, [46]).

## 3. Results

### 3.1. T1 Hyperintensity in Early Postoperative MRI and 3D Superimposition

**Figure 3:**
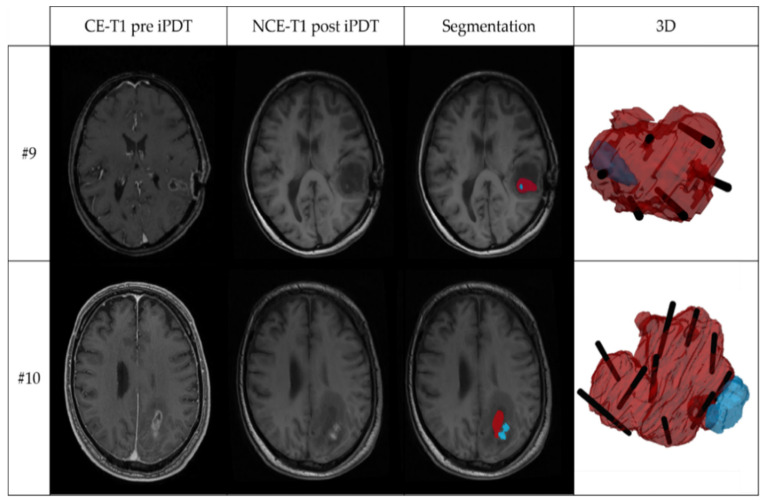
Two examples of MRI and 3D superimposition visualization, showing: contrast-enhanced T1-weighted MRI before iPDT (CE-T1 pre iPDT); non-contrast-enhanced T1-weighted MRI after iPDT (NCE-T1 post iPDT); segmentation of the tumor volume (red) and intrinsic T1 hyperintensity (blue) based on the CE-T1 pre iPDT and NCE-T1 post iPDT images; and 3D superimposition of tumor volume (red), intrinsic T1-hyperintensity volume (blue), and planned localization of irradiating tips of cylindrical diffuser fibers (black). The upper panel shows iPDT case #9 with the largest overlap coefficient (OV = 0.99); the lower panel iPDT case #10 with smallest overlap coefficient (OV = 0.02).

Intrinsic T1 hyperintensity was visible in early postoperative MRI (median: 22 h after the end of therapy; range: 15–29 h) after iPDT. The calculated geometric and overlay measures of the tumor volume (V_T_) and the intrinsic T1 hyperintensity volume (V_hyp_) are listed in Table 2. While the median T1 hyperintensity volume (V_hyp_) was 790.1 mm^3^, there was one case with V_hyp_ less than 100 mm^3^ and two cases with V_hyp_ larger than 1500 mm^3^. The maximum diameter of the intrinsic T1 hyperintensity ranged between 3 mm and 19.1 mm (median: 8.45 mm). The comparison between V_hyp_ and the tumor volume V_T_ based on V_hyp_/V_T_ shows that in 7/12 patients, the intrinsic T1 hyperintensity volume after treatment was larger than 5% of the tumor volume. In only three cases was it larger than 30%. With regard to (V_hyp_ ∩ V_T_)/V_T_, in only 5/12 cases more than 5% (max 28%) of the tumor volume was covered by T1 hyperintensity. In 11/12 cases V_hyp_ was smaller than V_T_ so that OV = (V_hyp_ ∩ V_T_)/V_hyp_. Thereof, in 7/11 cases, at least 60% of the intrinsic T1 hyperintensity are localized within the tumor (median: 0.61). In 3/12 cases, the volume of intrinsic T1 hyperintensity was almost completely located within the tumor volume (OV > 0.90). This is demonstrated in Figure 3, showing in the upper panel the T1-weighted MR images and segmentations for case #9 with the largest overlap, OV = 0.99. The 3D superimposition shows that the intrinsic T1 hyperintensity region (blue) is almost completely located within the contrast enhancing tumor region (red) and crossing one of the CDF sections (black). In the lower panel of Figure 3, case #10 with the smallest overlap, OV = 0.02, is shown. Here, the 3D superimposition shows that the intrinsic T1 hyperintensity volume is located almost entirely outside the segmented tumor volume, but next to two CDFs.

In total, 5/11 patients had an OV < 0.5, but the volume of intrinsic T1 hyperintensity was still located in close proximity to the contrast-enhancing tumor region. DSC and JSC show the positional agreement between tumor and T1 hyperintensity to be 0.39 or 0.24 at most, respectively (median: 0.09/0.05). A higher OV than DSC or JSC further indicates the high volume differences between V_T_ and V_hyp._ Further comparison of the intrinsic T1 hyperintensity and the position of CDF-pairs demonstrated that 298/392 CDF-pairs (76%) had a local intrinsic T1 hyperintensity involvement within the light transmission zone surrounding the CDF-pair. The residual 94/392 CDF-pairs (24%) showed no local intrinsic T1 hyperintensity involvement. Of all CDF-pairs, 108/392 (27.6%, or 36.2% of all 298 CDF-pairs with T1 hyperintensity) had >5% T1 hyperintensity volume in the light transmission zone surrounding the CDF-pairs.

### 3.2. Effects of Different Blood Volume Fractions on Laser Light Transmission at 635 nm

**Figure 4:**
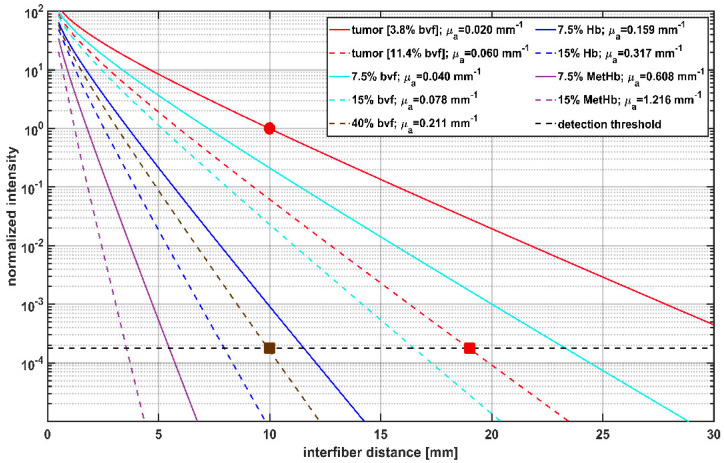
Calculation of transmitted light intensity at 635 nm between cylindrical diffuser fibers (CDFs) with a 2 cm diffuser length, and its dependency on the interfiber distances for different blood volume fractions (bvf) and hemoglobin species. Blood is approximated to contain 85% HbO_2_ and 15% Hb. Transmitted light intensities are normalized to tumor (μ_a_ = 0.02 mm^−1^, μs’ = 2 mm^−1^) at 10 mm interfiber distance (red circle). The legend shows the different blood volume fractions of hemoglobin species and the resulting μ_a_ assumed for the calculation with μ_s_’ set constant to 2 mm^−1^. The horizontal dashed line at 1.9 × 10^−4^ shows the estimated detection threshold of the iPDT SOM setup. The red square indicates that in case of a tumor with 11.4% bvf the detection threshold is reached at an interfiber distance of 19 mm. The brown square shows that for 40% bvf the detection threshold is already reached at an interfiber distance of 10 mm.

As intrinsic T1 hyperintensity in early postoperative MRI may be associated with blood degradation products, theoretical calculations were made to illustrate the effects of increasing blood volume fraction (bvf) and different hemoglobin species on the detected light intensities. The results shown in Figure 4 represent the calculated dependency of the treatment light transmission on the interfiber distance between two parallel CDFs with 20 mm diffuser length. The shown intensity values are normalized to the intensity in tumor tissue with optical parameters $\mu_{a}=0{.02\;\mathrm{mm}}^{-1};\mu_{s}^{\prime }=2\;{\mathrm{mm}}^{-1}$ (bvf = 3.8%, red solid line) at 10 mm interfiber distance indicated by a red circle in Figure 4. These values are compared to the detection threshold of the SOM setup obtained from the intraoperative data marked by a horizontal dashed line. A slightly increased bvf (7.5% or 15%, cyan curves), potentially induced by a hemorrhage, would reduce the signal intensity. However, transmitted laser light would still be detectable at interfiber distances up to at least 17 mm. In case the bvf increases further, e.g., if a hemorrhage is induced during fiber placement before iPDT illumination, the absorption increases further and leads to a steeper signal decay (brown dashed curve). According to the calculations and assumptions, a bvf of at least 40% accounts for absorption, making it impossible to detect a transmitted light signal for interfiber distances larger than 10 mm (brown square). In addition to changes in bvf, spectral changes of the absorbing molecules may occur. For example, with ongoing iPDT and deoxygenation of the blood, Hb might become the relevant absorber. With Hb as the absorber, the effect of the increase on μ_a_ would rise by a factor of four, resulting in a further reduction of transmitted light intensity, as shown by the dark blue lines (Hb = 7.5%/15%). A fraction of about 10% Hb would have a similar effect as a hemorrhage with 40% bvf. The occurrence of methemoglobin would lead to a further drastic reduction of signal intensity (violet curves). A MetHb fraction of 15% in tissue (μ_a_ (15% MetHb) = 1.2 mm^−1^) would lead to a complete loss of detectable laser light for CDF separations larger than 4 mm (violet dashed curve). Calculations show that 1% bvf of MetHb would lead to an absorption of μ_a_ ~ 0.08 mm^−1^, which is four times higher than the preset ‘normal’ tumor absorption of μ_a_ = 0.02 mm^−1^ with a bvf of 3.8%. Thus two different changes must be considered for interpretation of measured SOM data: changes in the bvf and changes in the absorption spectrum due to photo-induced molecular processes.

### 3.3. Intraoperative Transmission Intensity Change and Its Comparison to Intrinsic T1 Hyperintensity

**Figure 5:**
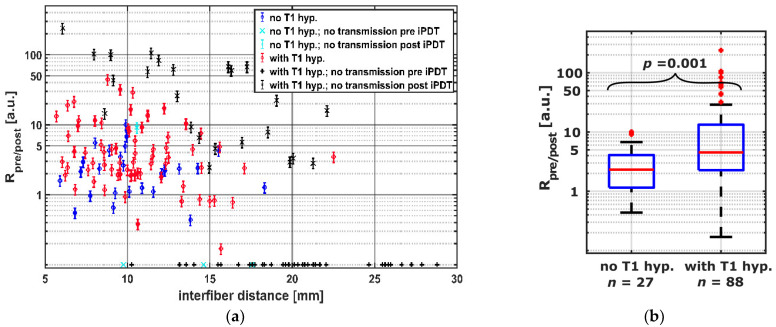
(**a**) Transmission ratios R_pre/post_ for all CDF-pairs compared to interfiber distance. The colors and symbols indicate whether transmission was detectable post iPDT and whether T1 hyperintensity was observed (see legend). Crosses near the x-axis indicate CDF-pairs with no detectable treatment light transmission at iPDT start, for which R_pre/post_ is defined as 0. (**b**) Comparison of CDF-pairs with and without local intrinsic T1 hyperintensity, considering only CDF-pairs with detectable transmission pre iPDT and interfiber distance ≤ 19 mm.

Intraoperative SOM data were acquired for 160 CDF-pairs. For 132/160 CDF-pairs, the interfiber distance was less or equal to 19 mm, the distance at which a signal should be detectable even with the highest absorption measured for undisturbed “brain adjacent tumor” tissue (μ_a_ = 0.06 mm^−1^, red square in Figure 4) [47]. Table 3 shows the numbers of CDF-pairs with detectable transmitted laser light and PpIX fluorescence pre and post iPDT and with or without local intrinsic T1 hyperintensity in the light transmission zone surrounding a CDF-pair. As CDF-pairs with interfiber distances larger than 19 mm may have no or very low laser light transmission even at ‘normal’ optical tissue properties, only CDF-pairs with interfiber distances ≤19 mm were included in the statistical evaluations (132/160). Of these 132 CDF-pairs, 17 showed no detectable laser light transmission at iPDT start. The 17 CDF-pairs divide into 14 cases with and 3 cases without local intrinsic T1 hyperintensity in early postoperative MRI, with a significantly higher number of those with local intrinsic T1 hyperintensity (*p* = 0.008). After iPDT, additional 25 CDF-pairs (increase from 39 to 64) had no detectable laser light transmission, which includes additional 20 CDF-pairs with interfiber distance ≤19 mm (increase from 17 to 37). Thereof 1 CDF-pair had no local intrinsic T1 hyperintensity, and 19 had local intrinsic T1 hyperintensity involvement. When considering CDF-pairs with interfiber distance ≤19 mm, PpIX fluorescence could be measured for 95/115 CDF-pairs pre iPDT, of which 72/95 had a local intrinsic T1 hyperintensity. After iPDT, residual PpIX fluorescence was observed in neither iPDT case nor for any CDF-pair. Two iPDT cases (#6 and #11) unite 19/20 (95%) of all CDF-pairs without detectable PpIX fluorescence pre–iPDT, corresponding to all CDF-pairs of these two cases. In Figure 5a, the calculated intra-operative pre versus post iPDT treatment light transmission ratio R_pre/post_ is plotted against the interfiber distance. The data are grouped by CDF-pairs with local intrinsic T1 hyperintensity involvement (red and black symbols) and those without (blue and cyan symbols). R_pre/post_ values larger than 50 were observed only for CDF-pairs with detectable laser light transmission signal before but not after iPDT and with local intrinsic T1 hyperintensity in early postoperative MRI (black symbols). In total, 9/160 (6%) CDF-pairs had a R_pre/post_ smaller than 0.8 (increase of *I*_t_ > 20%; maximum error of R_pre/post_ is 20%). For 98/160 CDF-pairs, R_pre/post_ was larger than 1.20 (decrease of *I*_t_ > 20%). The comparison of CDF-pairs with detectable transmission pre iPDT in Figure 5b shows that the ratio R_pre/post_ is larger for CDF-pairs with local intrinsic T1 hyperintensity than for those without (median/interquartile range: 4.5/[2.3, 13.3] versus 2.3/[1.2, 4.1], *p* = 0.001).

**Figure 6:**
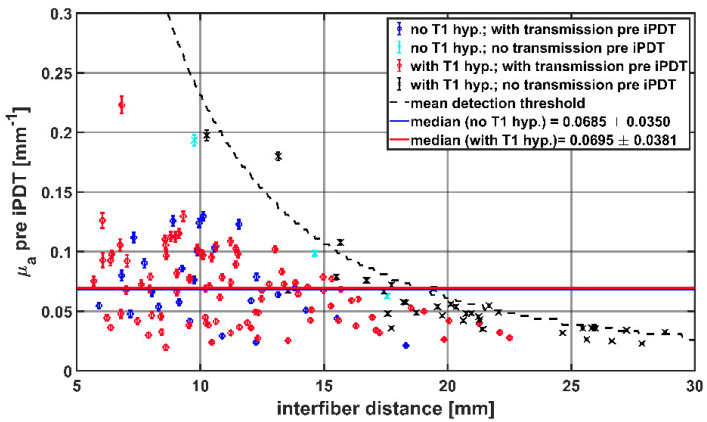
Calculated μ_a_ values before start of iPDT compared to interfiber distance. The colors and symbols indicate whether transmission was detectable pre iPDT and whether intrinsic T1 hyperintensity was observed (see legend). The dashed line represents the mean detection threshold of the SOM setup.

The average μ_a_ of the tissue between two CDFs, calculated from the SOM measurements pre iPDT, are shown in Figure 6 as a function of the interfiber distance. The median μ_a_ pre iPDT determined from all SOM measurements with detectable transmission signals (blue and red symbols) was 0.068 mm^−1^ (interquartile range: [0.045, 0.093]). There was no significant difference between the μ_a_ values for CDF-pairs with and without intrinsic T1 hyperintensity (red versus blue symbols): 0.069 mm^−1^ vs. 0.070 mm^−1^; *p* = 0.37.

**Figure 7:**
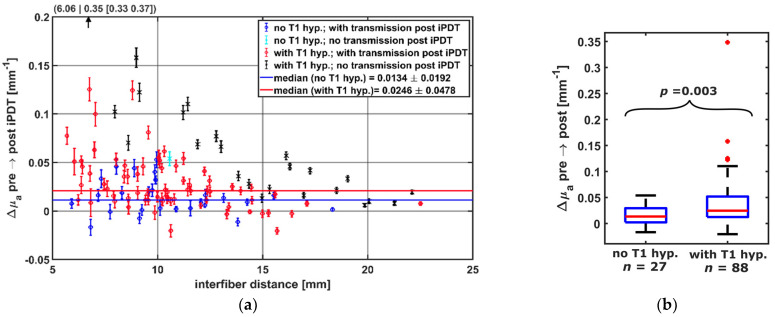
(**a**) Calculated Δμ_a_ dependent on the interfiber distance between CDF-pairs. Colors indicate whether transmission was detectable post iPDT and whether intrinsic T1 hyperintensity was observed (see legend). For better visibility, the data point at (6.06|0.35) is omitted. (**b**) Comparison of Δμ_a_ of CDF-pairs with and without local intrinsic T1 hyperintensity, considering only pairs with transmission pre iPDT and interfiber distance ≤19 mm.

The calculation of Δμ_a_ showed (see Figure 7a) that only for CDF-pairs with local intrinsic T1 hyperintensity involvement (red, black), a Δμ_a_ greater than 0.054 mm^−1^ was observed. A decrease in μ_a_ was seen for 12 CDF-pairs. Of these, 8 had local intrinsic T1 hyperintensity involvement. Considering only CDF-pairs with an interfiber distance ≤19 mm, Δμ_a_ was higher when intrinsic local T1 hyperintensity was observed (median/interquartile range in mm^−1^: 0.025/[0.012, 0.052] versus 0.0134/[0.0022, 0.030], *p* = 0.003, Figure 7b). CDF-pairs with no detectable treatment light transmission at iPDT start were not considered, as no Δμ_a_ could be calculated.

### 3.4. Comparing Intrinsic T1 Hyperintensity Strength

**Figure 8:**
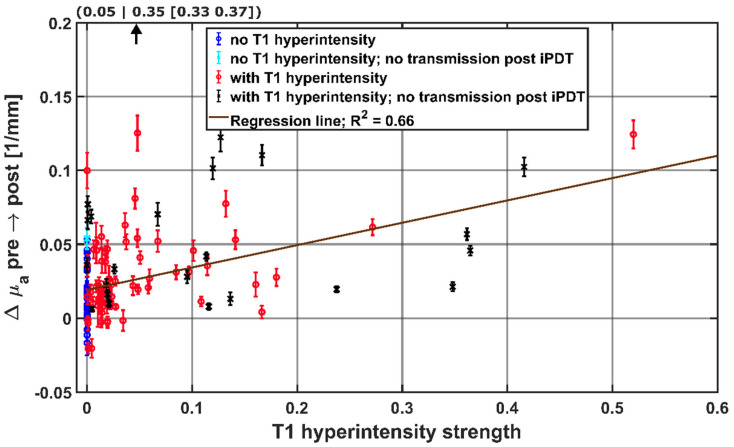
Hyperintensity strength in the light propagation volume surrounding CDF-pairs, with the calculated regression line obtained by the Spearman test. For better visibility, the data point at (0.05|0.35) is not shown. The colors and symbols indicate whether transmission was detectable post iPDT and whether intrinsic T1 hyperintensity was observed (see legend).

The signal intensity of the intrinsic T1 hyperintensity and the degree of overlap between intrinsic T1 hyperintensity and light transmission zone were evaluated to investigate whether a stronger involvement of T1 hyperintensity for a given CDF-pair leads to a stronger influence on the observed absorption change Δμ_a_. For this purpose, the fraction of T1 hyperintensity within the light transmission zone between a CDF-pair was multiplied by I_T1_. The resulting T1 hyperintensity strength was compared to Δμ_a_ in Figure 8, separately for each CDF-pair and with respect to detectable laser light transmission at the end of iPDT. The linear two-sided Spearman regression (R² = 0.66, *p* < 0.001, *N* = 115) indicates that for higher Δμ_a_ values, a higher intrinsic T1 hyperintensity strength can be expected in the light transmission zone. From CDF-pairs without local intrinsic T1 hyperintensity (blue and cyan symbols, T1 hyperintensity strength = 0), the degree of T1 hyperintensity strength that leads to a significant increase in Δμ_a_ can be deduced. According to Figure 8, a T1 hyperintensity strength of approximately 0.20 leads to an average Δμ_a_ of 0.05 mm^−1^.

### 3.5. Analysis of PpIX Fluorescence

Analyzing Δμ_a_ in relation to the occurrence of PpIX fluorescence between a CDF-pair showed, for the 20 CDF-pairs without local fluorescence before iPDT, that either an increase (11/20) or a decrease (9/20) in μ_a_ could be observed (Δμ_a_ range: −0.021 mm^−1^ to 0.125 mm^−1^). A Δμ_a_ < −0.005 mm^−1^ only occurred for CDF-pairs with no PpIX fluorescence before iPDT. Overall, for CDF-pairs without detectable fluorescence, the median Δμ_a_ is more than 2.7 times smaller compared to CDF-pairs with detectable fluorescence before iPDT. This indicates a possible relationship between higher Δμ_a_ and the occurrence of fluorescence, but the difference is not statistically significant (0.0098 mm^−1^ vs. 0.0265 mm^−1^, *p* = 0.063). Only CDF-pairs with detectable PpIX fluorescence had intrinsic T1 hyperintensity strengths larger than 0.14.

## 4. Discussion

Analyzing intraoperative SOM measurements from patients receiving iPDT for high grade glioma, a decrease in transmitted treatment light related to an increase in tissue absorption for 85% of the evaluated CDF-pairs could be observed for the treatment wavelength of 635 nm. On postoperative MRI, areas with intrinsic T1 hyperintensity in the treatment area were identified and analyzed with respect to their location relative to the iPDT treatment volume. A stronger increase in μ_a_ correlates significantly with the occurrence of intrinsic T1 hyperintensity in the light transmission zone surrounding a CDF-pair. No correlation between tissue absorption at iPDT start and intrinsic T1 hyperintensity in early postoperative MRI was observed. Further SOM analysis demonstrated a tendency towards higher Δμ_a_ related to if PpIX fluorescence was observed between a CDF-pair.

Intercranial intrinsic T1 hyperintensity may be caused by different substances, including MetHb, melanin, lipids, proteins, minerals, and others [48]. MetHb is a blood degradation product that could be procedure-related, produced with a potentially accelerated conversion due to iPDT [49].

High-grade gliomas form an especially pronounced capillary system with thinner vessel walls than normal blood vessels [50]. Silent, asymptomatic hemorrhages are reported in 20–60% of biopsies [51,52], albeit before MRI was readily available for trajectory planning. A small proportion of these silent hemorrhages were distant from the biopsy location itself and, therefore, described as trajectory-related. The occurrence of trajectory-related hemorrhages was confirmed by Casanova et al. [53,54], who even described injuries occurring at some distance from the trajectory. The diameters of intrinsic T1 hyperintensities found in the case of this study (3 mm to 19 mm) were of similar size to that of the silent hemorrhages found for biopsies (diameters < 5 mm to 40 mm) [51,52]. Clinically silent hemorrhages may have been treatment-related during iPDT and become visible as intrinsic T1 hyperintensity in early postoperative T1-weighted MRI due to the accelerated conversion of Hb species to MetHb by iPDT illumination. This accelerated conversion would be well consistent with findings in liquid tissue phantoms [23].

Some intrinsic T1 hyperintensity was found in every post iPDT non-enhanced T1-weighted sequence analyzed in the presented cohort. In most iPDT cases, either the overlap (OV) of the intrinsic T1 hyperintensity with the tumor volume was high (above median), or the volume of intrinsic T1 hyperintensity was very small (below median). Therefore, the observed T1 hyperintensity was mainly confined within the tumor volume or directly adjacent to the contrast enhancing tumor margin. Unfortunately, the contrast enhancement in MRI does not fully represent the actual tumor volume, as contrast enhancement is mainly related to the breakdown of the blood brain barrier but does not cover the infiltration zone [44,55]. It is known that FET-PET allows better visualization of the metabolically active tumor, and, in most cases, high-grade gliomas show a larger volume in FET-PET imaging compared to MRI [56]. This is consistent with reports stating that PpIX is also accumulated in the diffuse infiltration zone or peritumoral zone with metabolically active tumor cells [31,57]. To take into account that MRI does not display the full tumor volume, the iPDT treatment planning is carried out in such a way that the PDT effect is extended into the PpIX-accumulating infiltration zone. For this purpose, the peripheral CDFs are placed within the contrast-enhanced tumor volume but close to the tumor margin. Taking both aspects into account, it can be assumed that the OV coefficients between the T1 hyperintensity volume and the real tumor volume, including the infiltration zone not visible in contrast-enhanced MRI, are larger than those OV coefficients calculated only based on the contrast-enhanced MRI. Thus, it can be concluded that all T1 hyperintensity volumes observed in this work are located within the full tumor volume, including the infiltration zone, and within the intended iPDT treatment volume. A notable exception was iPDT case #4, which was treated twice. In the second treatment, case #4b, a four-fold smaller tumor volume was targeted compared to the first treatment. About 2/3 of the intrinsic T1 hyperintensity volume recorded after the second iPDT overlapped with the tumor volume treated in the first iPDT session. In this case, the formation of an additional fraction of MetHb or another intrinsically T1 hyperintense substrate between the two iPDT sessions can be assumed.

The occurrence of a hemorrhage may by itself immediately cause an absorption increase due to the increased bvf in the tissue. However, as observed in the results, this would probably not lead to a complete loss of transmission between some CDF-pairs. In case of a reference interfiber distance of 10 mm, capillary blood would need to occupy 40% of the tissue volume to reduce the light transmission to below the detection limit. As a further effect, spectral changes due to potentially photo-induced molecular changes must be taken into account. If oxygen consumption by the iPDT procedure led to the formation of deoxygenated hemoglobin, a bvf of about 10% would be sufficient for a total loss of transmission. The formation of MetHb would further increase the absorption coefficient at an iPDT treatment wavelength of 635 nm, and even small bvf would have a severe effect on transmission. Deoxygenation of blood is well consistent with previous findings on liquid phantoms [23]. In addition, a directly ROS-induced MetHb formation has indeed been reported [36,58,59,60]. PDT-induced hemoglobin deoxygenation may be the probable cause in all instances, where a high increase in tissue absorption was observed, and ROS-induced MetHb-formation can also not be excluded.

By calculating the intrinsic T1 hyperintensity strength, better quantification of the local T1 hyperintensity volume’s influence on the light propagation between a CDF-pair should be achieved to correlate it with a potentially iPDT-induced absorption change. This was only partly successful, as the conclusion that a strong and large T1 hyperintensity is only observed when the absorption increase during iPDT was high is not very well-founded with R² = 0.66. Furthermore, it was observed that the T1 hyperintensity strength was relatively small between many CDF-pairs, although a high Δμ_a_ was calculated. With the observed Δμ_a_ being higher than expected if only induced by oxygenated blood, hemoglobin deoxygenation, or even MetHb production by iPDT have to be considered.

Assuming that CDF insertion leads to an intercranial hemorrhage, this would usually be followed by a hyperacute stage, during which predominantly HbO_2_ is expected in the tissue, followed by an acute stage, where Hb is formed within 24−48 h after onset of the hemorrhage. Only after 2−7 days, intrinsic T1 hyperintensity is usually expected as a consequence of the MetHb formation [24,61]. Due to oxygen consumption during the iPDT procedure, hemoglobin deoxygenation may occur more rapidly, and MetHb formation may be accelerated. Thus, the intrinsic T1 hyperintensity occurring already one day after iPDT could be explained.

With the observed intrinsic T1 hyperintensity and increase in tissue absorption, possible detrimental effects on the iPDT efficacy need to be addressed. With increasing tissue absorption, the initial light dosimetry may become invalid, potentially leading to under-treatment. While increased absorption leads to decreased light penetration depth, the implications for the treatment depth are not so clear because photobleaching of PpIX is a further decisive factor. Due to the high light dose applied, complete photobleaching—and, thus, induced tissue damage—can be achieved. Indeed, there is no case in the performed SOM measurements where residual fluorescence was found after iPDT—independent of CDF separation, pre iPDT absorption, or absorption change. Of course, when there is no detectable treatment light transmission signal before iPDT, a lack of fluorescence signal cannot be interpreted as a lack of PpIX. However, in all cases with a detectable treatment light transmission signal after iPDT, complete PpIX photobleaching could be confirmed. In the cases with treatment light transmission signal before but without treatment light transmission signal after iPDT, more detailed time-dependent SOM recordings would be required to unravel when during the treatment absorption increase and fluorescence decrease occurred. Overall, it can be expected that there will be an effective light dose applied throughout the entire contrast enhanced tumor volume. However, as far as penetration into the infiltration zone outside the contrast-enhanced tumor volume is concerned, higher absorption would lead to a smaller necrosis depth.

The induction of necrosis requires PpIX activation and oxygen consumption for ROS generation. One may speculate that the estimated absorption increase in tissue should be higher for CDF-pairs with detectable PpIX fluorescence signal at iPDT start and subsequent local intrinsic T1 hyperintensity. Indeed a tendency towards a stronger absorption increase in the tissue for these CDF-pairs could be observed. Furthermore, the results also indicated that for CDF-pairs with no PpIX fluorescence, the increase in tissue absorption is smaller, or even a decrease may happen. Undetectable PpIX fluorescence does not necessarily mean that no PpIX was accumulated in the tumor, as the detected PpIX fluorescence signal depends on the localization of the PpIX relative to the CDF-pair and on tissue inhomogeneities inducing inhomogeneous optical tissue properties. So, there is the possibility of a sufficient PpIX amount present in the tumor for iPDT but not causing detectable PpIX fluorescence pre iPDT.

For the quantitative analysis of optical tissue properties, absolute values of the absorption coefficient μ_a_ were calculated based on the diffusion approximation of the radiative transfer equation. Unlike the directly measured treatment light transmission signals, the computed values for μ_a_ are independent of interfiber separation. The applied diffusion approximation assumes homogeneous optical tissue properties, so the possibility of individual μ_a_ values in different tissue regions is disregarded. Thus, only an averaged μ_a_ value could be computed for the volume surrounding a CDF-pair [33]. This affects the comparability of the obtained μ_a_ values (range: 0.02–0.22 mm^−1^) with optical properties of brain tissue (white matter, grey matter, glioblastoma) in the literature (range 0.02–0.08 mm^−1^) [62,63,64]. Therefore, the obtained results might be overestimated due to the assumption of a constant μ_s_’ = 2 mm^−1^, so differing μ_s_’ values for brain tissue (range 1.0–6.0 mm^−1^) are neglected.

Finally, it must be mentioned that the data amount was limited due to the low number of iPDT cases. Further data had to be excluded due to sophisticated technical demands issues for this analysis, e.g., incorrectly measured spectral data or MRI sequences that were not comparable to the others. In some iPDT cases, SOM was only performed for a selection of CDF-pairs, to decrease measurement time and, therefore, minimize patient load. A revised, dedicated iPDT protocol with optimized equipment might allow to speed up the SOM data acquisition process and increase the available data amount. In addition, these technical and clinical constraints limit the statistical power of the data. The calculated p-values, even if they are low, should be carefully interpreted as only indicating tendencies. To obtain results with stronger significance, future analyses on larger patient collectives are necessary, ideally using improved automated SOM concepts.

The overall aim of the presented investigation and future work should be to derive additional information from different monitoring applications (e.g., SOM, MRI, PET) by combining them and comparing abnormalities to further improve iPDT procedure, both clinically and technically. This also contributes to gain more detailed insight into the phototoxic reactions in the target tissue. With enhanced interpretation of the recorded spectral and MRI data sets, the benefit of the patients suffering from this kind of cancer with a bad prognosis may be further improved.

## 5. Conclusions

SOM measurements enable an individual intra-operative assessment of absorption and fluorescence. With calibrated detectors, known trajectory coordinates, and laser light powers, one can calculate optical parameters of perfused viable tumor tissue in situ, at least in the form of mean values, albeit their detailed spatial distribution cannot be derived. This should lead to a much more reliable database of optical properties of GBM tissue, in particular for in vivo interventions. If optical parameters in individual patients or locations should turn out to strongly deviate from the mean values, a personalized irradiation time might at least partly compensate for this condition.

Frequently, an increase in absorption between measurements before and after iPDT was observed, which correlated with early postoperative intrinsic T1 hyperintensity in non-contrast-enhanced T1-weighted MRI data. This may indicate that clinically silent hemorrhage was induced during cylindrical diffuser fiber insertion. During iPDT, a ROS-driven accelerated hemoglobin deoxygenation and conversion to MetHb may then occur and may impair the irradiation efficacy. However, the fraction of tumor volume affected by T1 hyperintensity was below 28% in all cases (<10% in most cases). Substantial PDT-related effects will thus be induced in the tumor region, even in non-ideal situations.

On the basis of these results, additional evaluations and research should allow for further elucidation on the mechanisms of iPDT-related changes in tissue absorption and intrinsic T1 hyperintensity.

## Figures and Tables

**Table 1:** Irradiation and spectral online monitoring characteristics, displaying the number of measured spectra and used cylindrical diffuser fibers (CDFs) with the irradiation parameters during the treatment.

iPDT Case	Total Number of Measured Spectra	CDFs Used	Median Interfiber Distance	CDF Power	Irradiation Time	Total Applied Light Dose
			[mm]	[mW/cm]	[s]	[J]
1	12	4	10.0	200	3600	8640
2	28	7	13.2	200	5400	20,520
3	30	6	10.5	200	3600	8640
4a	12	4	10.2	200	3600	8640
4b	2	2	10.0	200	3600	2880
5	20	5	11.0	100	7200	7200
6	20	5	12.3	150	7200	10,800
7	42	7	10.6	200	3600	12,960
8	72	9	13.0	200	3600	15,120
9	30	6	11.0	200	3600	12,960
10	32	10	13.2	200	3600	18,720
11	20	5	11.4	133	5400	8694

**Table 2:** Tumor volume (V_T_) and properties of the intrinsic T1 hyperintensity volume (V_hyp_) of the different iPDT cases and the calculated Overlap (OV), Dice (DSC), and Jaccard (JSC).

iPDT Case	$V_{T}$	$V_{\mathrm{hyp}}$	$\mathrm{Max}.\;\mathrm{Diameter}\;V_{\mathrm{hyp}}$	$I_{T1}$	$\frac{V_{\mathrm{hyp}}}{V_{T}}$	$\frac{V_{\mathrm{hyp}}\cap V_{T}}{V_{T}}$	OV	DSC	JSC
	(mm^3^)	(mm^3^)	(mm)	a.u.	(%)	a.u.	a.u.	a.u.	a.u.
1	1382	50	4.6	0.67	3.7	0.02	0.42	0.03	0.02
2	8574	3928	19.1	1.45	45.8	0.28	0.62	0.39	0.24
3	1955	544	9.6	0.80	27.8	0.01	0.03	0.01	0.01
4a	2269	1740	12.0	0.56	76.7	0.22	0.29	0.25	0.14
4b	537	978	15.0	0.91	182.1	0.03	0.03	0.02	0.01
5	4079	137	4.5	0.59	3.4	0.02	0.66	0.04	0.02
6	6920	152	4.4	0.71	2.2	0.02	0.96	0.04	0.02
7	11,020	361	3.0	0.73	3.3	0.03	0.94	0.06	0.03
8	13,650	1374	10.0	0.81	10.1	0.08	0.79	0.14	0.08
9	7176	115	5.1	0.73	1.6	0.02	0.99	0.03	0.02
10	15,340	1065	13.1	1.19	6.9	0.00	0.02	0.00	0.00
11	3990	603	7.3	0.74	15.1	0.09	0.60	0.16	0.09
median	6064	790	8.45	0.74	12.8	0.05	0.61	0.09	0.05

**Table 3:** Overview of evaluated CDF-pairs and observed detectability of treatment light transmission intensity (transmission) or PpIX fluorescence signal before (pre iPDT) and after (post iPDT) conducted iPDTs.

Number of CDF-Pairs	Total	Transmission Detectable				Fluorescence Detectable			
		Pre iPDT		Post iPDT		Pre iPDT		Post iPDT	
		yes	no	yes	no	yes	no	yes	no
All interfiber distances	160	121	39	96	64	114	46	0	160
Interfiber distance ≤19 mm ^1^	132	115	17	95	37	95 ^2^	20 ^2^	0	115 ^2^
With local intrinsic T1 hyperintensity	102	88	14	69	33	72 ^2^	16 ^2^	0	88 ^2^
Without local intrinsic T1 hyperintensity	30	27	3	26	4	23 ^2^	4 ^2^	0	27 ^2^

^1^ Only CDF-pairs with interfiber distance ≤19 mm were included in statistical evaluations to avoid bias by spectra with undetectable transmission at “normal” optical tissue properties (μ_a_ ≤ 0.06 mm^−1^). ^2^ Transmission signal detectable pre iPDT (in total 115 CDF-pairs).

## Data Availability

No new data were created or analyzed in this study. Data sharing is not applicable to this article.
